# Determinants of pre-eclampsia/Eclampsia among women attending delivery Services in Selected Public Hospitals of Addis Ababa, Ethiopia: a case control study

**DOI:** 10.1186/s12884-017-1507-1

**Published:** 2017-09-15

**Authors:** Teklit Grum, Abiy Seifu, Mebrahtu Abay, Teklit Angesom, Lidiya Tsegay

**Affiliations:** 1 School of Public Health, College of Health Sciences, Aksum University, Aksum, Ethiopia; 2 School of Public Health, College of Health Sciences, Addis Ababa University, Addis Ababa, Ethiopia; 3 School of Nursing, College of Health Sciences, Aksum University, Aksum, Ethiopia

**Keywords:** Determinants, Pre-eclampsia/Eclampsia, Women, Ethiopia

## Abstract

**Background:**

Pre-eclampsia is a pregnancy-specific hypertensive disorder usually occurs after 20 weeks of gestation. It is one of the leading causes of maternal and perinatal morbidity and mortality worldwide. In Ethiopia, the major direct obstetric complications including pre-eclampsia/eclampsia account for 85% of the maternal deaths. Unlike deaths due to other direct causes, pre-eclampsia/ eclampsia related deaths appear to be increasing and linked to multiple factors, making prevention of the disease a continuous challenge. The aim of this study is to assess determinants of pre-eclampsia/eclampsiaamong women attending delivery services in selected public hospitals in Addis Ababa, Ethiopia.

**Methods:**

Hospital based unmatched case control study design was employed. The study wasconducted in Addis Ababa among women attending delivery services in two public hospitals from December, 2015 G.C. to February, 2016 G.C. with sample size of 291 (97 cases and 194 controls). Women with pre-eclampsia/eclampsia were cases and women who had not diagnosed for pre-eclampsia/eclampsia were controls. Case-control incidence density sampling followed by interviewer administered was conducted using pretested questionnaire. The data was entered in Epi Info 7 software and exported to STATA 14 for cleaning and analysis. Descriptive statistics were used todisplay the data using tables compared between cases and controls. To compare categorical variables between cases and controls Chi-squared testwas used. Both bivariable and multivariable logistic regression analyses were computed to identify the determinants of pre-eclampsia/eclampsia.

**Results:**

Factors that were found to have statistically significant association with pre-eclampsia or eclampsia were primigravida (AOR: 2.68, 95% CI: 1.38, 5.22), history of preeclampsia on prior pregnancy (AOR: 4.28, 95% CI: 1.61, 11.43), multiple pregnancy (AOR: 8.22, 95% CI: 2.97, 22.78), receiving nutritional counseling during pregnancy (AOR: 0.22, 95% CI: 0.1, 0.48) and drinking alcohol during pregnancy (AOR: 3.97, 95% CI: 1.8, 8.75).

**Conclusions:**

The study identified protective and risk factors for pre-eclampsia/eclampsia. To promptly diagnose and treat pre-eclampsia, health workers should give special attention to women with primigravida and multiple pregnancy. Besides, health care providers should provide nutritional counseling during ANC, including avoiding drinking alcohol during their pregnancy.

**Electronic supplementary material:**

The online version of this article (10.1186/s12884-017-1507-1) contains supplementary material, which is available to authorized users.

## Background

Pre-eclampsia is a pregnancy-specific hypertensive disorder usually occurs after 20 weeks of gestation. It is a rapidly progressive condition characterized by elevated blood pressure and protein in the urine [1]. If undetected early, it can lead to eclampsia which is severeand one of the top five direct causes of maternal and infant adverse outcome [1, 2].

The exact cause of pre-eclampsia/eclampsia remains unclear. However, abnormally implanted placenta is considered to be major predisposing. This abnormally implanted placenta is believed to result in poor uterine and placental perfusion, which results a state of hypoxia and increased oxidative stress and the release of anti-angiogenic proteins into the maternal plasma along with inflammatory mediators into the maternal plasma [3].

Pre-eclampsia/eclampsia is one of the leading causes of maternal and perinatal morbidity and mortality worldwide. It is an important cause of severe morbidity, long term disability and death among both mothers and their babies. In Africa and Asia, nearly one tenth of all maternal deaths are associated with hypertensive disorders of pregnancy, whereas one quarter of maternal deaths in Latin America have been associated with pre-eclampsia/eclampsia complications [3]. The majority of deaths due to pre-eclampsia/eclampsia are avoidable through the provision of timely and effective care to the women presenting with these complications. Optimizing health care to prevent and treat women with hypertensive disorders is a necessary step towards reducing maternal and infant mortality and morbidity [4].

In Ethiopia the major direct obstetric complications (hemorrhage, obstructed labor, pre-eclampsia/eclampsia, unsafe abortion, sepsis) account for 85% of the maternal deaths as well as many acute and chronic illnesses [5]. Among the five major causes of maternal death severe pre-eclampsia/ eclampsia(PE/E) accounts 11% [6]. The country has identified PE/E as one of the major causes of maternal mortality and is working on improvement of the main components of quality health services. These include capacity-building (ensuring pre-service and in-service training for health providers to detect and manage PE/E, including magnesium sulfate in the national obstetric service guidelines), logistics (making supplies available to health facilities for management of PE/E), and supportive supervision and mentorship with the Obstetrics & Gynecological Society and other partners. Nevertheless, the late antenatal care initiation and limited evidences on causes for pre-eclampsia made difficult on controlling it. As a result, it remains a serious and poorly understood complication of pregnancy, which can progress to maternal death [7].

Unlike maternal deaths due to other direct causes, pre-eclampsia/ eclampsiarelated deaths appear to be increasing and remains a major problem both in maternal and infants morbidity and mortality [8].Giving attention to such causes of maternal mortality could hasten the reduction of maternal mortality. But the determinants of pre-eclampsia/eclampsia have not been well documented in Ethiopia. Therefore, the aim of this study is to assess determinants of pre-eclampsia/eclampsia.

## Methods

### Study setting

Hospital based unmatched case control study design was conducted in Addis Ababa in two selected public hospitals from December, 2015 G.C. to February, 2016 G.C.The source population for the study was all women attending delivery service in Gandhi and Zewditu Memorial Hospitals. The study population was the selected pregnant women attending delivery services in both hospitals.

### Inclusion criteria

The cases were women with blood pressure ≥ 140 mmHg systolic or ≥90 mmHg diastolic on two separate readings taken at least four hours apart with previously normal blood pressure and when proteinuria is greater than or equal to 300 mg per 24-h urine collection or dipstick reading of 1+ after 28 weeks’ gestation [1]. The diagnosis involves history taking, physical examination and laboratory test. Cases were selected after the physicians made diagnosis as part of routine case for the women. The women’s charts were reviewed, and women were included as cases if they fulfilled the aforesaid diagnostic criteria.

Controls were women who were attending delivery care from both hospitals and were not diagnosed as pre-eclampsia/eclampsia. All cases and controls were seen by obstetrician in the hospitals.

### Exclusion criteria

Women with known hypertension and renal disease were excluded from the study. Even though the design was unmatched case control study, women who were not from Addis Ababa were excluded from both groupsto make it comparable. Women with serious medical conditions and who couldn’t give consent were also excluded from the study.

### Sample size determination

EPI INFO 7 was used to calculate the sample size using double population proportion formula by assuming primigravida as a risk factor with lowest odds ratio of 2.16 and 39% among controls from the literatures reviewed [9]. With assumptions 95% confidence interval, 5% marginal error and 80% power, the calculated sample size was 264. Considering 10% possible non-respondents final sample size of 291(97 cases and 194 controls) was estimated.

### Sampling procedure

Zewditu and Gandhi Memorial Hospitals were purposely selected because these two Hospitals are the largest public Hospitals with maternal health service under the Addis Ababa city administration. All cases who came for delivery services consecutively to both hospitals were included until the required sample size was fulfilled. Thestudy used an incidence density sampling approach to selecttwo control women who came for delivery services in the same facilities for each case. Sample size was split between the two hospitals proportional to their delivery caseload. According to the 2014/15 G.C HMIS report, GMH has annual delivery of 5984 while ZMH has annual delivery of 4440.

- Total annual delivery in both Hospitals = 5984 + 4440 = 10,424
- So, the sample size allocated for each hospital was;
- For GMH = 5984*291/10424 = 167 (56 cases and 111 controls)
- For ZMH = 4440*291/10424 = 124(41 cases and 83 controls)

Accordingly 167 and 124 samples were assigned for GMH and ZMH, respectively.

### Data collection procedure

Data was collected using pretested interviewer administered questionnaire (Additional file 1). Respondent’s medical charts were also reviewed to extract clinical laboratory results. The questionnaire was written in English, translated into Amharic (national language), and then translated back into English to assure its consistency. Four Amharic speaking diploma level midwife nurses were hired as data collectors. Two supervisors with BSc inmidwife nurses supervised the data collection and the principal investigator coordinated them. The supervisors daily checked the consistency, clarity and completeness of the collected questionnaires.

### Data analysis procedure

The collected questionnairewas checked manually and entered the data in to epi Info 7 software and exported to stata14 for cleaning and further analysis. Frequency run was made to check for accuracy, consistencies, missed values and variables. Descriptive statistics was used to describe the study populations using measures of frequency, central tendency and dispersion that was displayed using tables compared between cases and controls. To compare categorical variables between cases and controls Chi-squared testwas used.

Bivariable logistic regression analysis was used and variables with *P*-value < 0.05 in the Bivariable logistic regression analysis were considered for inclusion in to the multivariable logistic regression analysis to control the effect of confounders.Forwards selection strategy was used to include the variables in the multivariable model. Multicollinearity was checked among the independent variables using variance inflation factor. The necessary assumption of logistic regression was checked using Hosmer and Lemeshow goodness of fit test statistics. Variables with *P*-value < 0.05 in the multivariable logistic regression analysis were considered as independently significant determinant of pre-eclampsia/eclampsia.

### Data quality management

To ensure data quality, questionnaire was pretested, 2 days training was provided to the data collectors and the data collection was supervised. The consistency of data collection among the data collectors was check using Cronbach alpha before data collection. During the data collection supervisors checked each questionnaire for its completeness, consistency and accuracy. Data collectors were informed to correct for any missing values or reconcile inconsistency before discharging the respondents from that hospital. In addition, during data entry range values and pattern analysis that comply with certain regular expression format pattern found in the attributes was used to ensure the quality.Missing value was handled using cases pairwise statistical technique during data analysis.

## Results

### Socio-demographic characteristics of study participants

From a total of 291 (97 cases and 194 controls) selected from both hospitals, 6(2 cases and 4 controls) refused to participate in the study. A total of 285(95 cases and 190 controls) women who came for delivery care service in GMH and ZMH completed the interview with response rate of 97.9% for both groups.

The mean age of cases 25.42(SD: ±5.33) was lower than that of the controls 27.96(SD: ±4.67). Seventy-four 74(77.89%) of the cases and 158(83.16%) of the controls were with in age group of 20-34 years.Thirty-nine (41.1%) of the cases and 62 (32.6%) of the controls belong to Amhara ethnicity. The dominant religion of the study participants was orthodox with 57(60%) of the cases. Concerning to marital status of study participants, 71 (74.7%) of the cases and 161(84.7%) of the controls were married or living together with their partner. With regards to their occupation, majority of the participants were housewives 47(49.5%) of the cases and 69(36.3%) of the controls.

The median house hold monthly income of study participants was $143 with minimum of $19 and maximum of $476 with range of $457. Forty-six (48.42%) of the cases and 104(54.74%) of the controls of house hold monthly income were above the median of the study subjects (Table 1).

**Table 1:** Socio-demographic characteristics of study participants in selected public hospitals of Addis Ababa, Ethiopia, 2016

Variables	Cases	Controls	Pearson chi^2^	*p*-value
	Number (%)	Number (%)		
Age				
< 20	11(11.58)	10(5.26)		
20-34	74(77.89)	158(83.16)	3.7066	0.157
≥ 35	10(10.53)	22(11.58)		
Mean(SD)	25.42(±5.33)	27.96(±4.67)		
Ethnicity				
Amhara	39(41.1)	62(32.6)	3.2019	0.525
Oromo	23(24.2)	56(29.5)		
Tigray	12(12.6)	29(15.3)		
Gurage	12(12.6)	30(15.8)		
Others	9(9.5)	13(6.8)		
Religion				
Orthodox	57(60)	106(55.8)		
Muslim	17(17.9)	47(24.7)	3.0962	0.377
Protestant	11(11.6)	25(13.2)		
Others	10(10.5)	12(6.3)		
Marital status				
Never married	13(13.7)	13(6.8)	4.6947	0.096
Married/Living together	71(74.7)	161(84.7)		
Others	11(11.6)	16(8.4)		
Occupation				
House wife	47(49.47)	69(36.32)		
Merchant	11(11.56)	41(21.58)	10.4362	0.015
Employee	26(27.37)	69(36.32)		
Student	11(11.56)	11(5.79)		
Educational status				
No formal education	10(10.5)	17(8.9)		
Primary	24(25.3)	41(21.6)	3.8222	0.431
Secondary	33(34.7)	70(36.8)		
Vocational/Technical	14(14.7)	43(22.6)		
Higher education	14(14.7)	19(10)		
Monthly house hold income				
< $143	49(51.58)	86(45.26)	3.0672	0.080
≥ $143	46(48.42)	104(54.74)		

Variables which had significant association with pre-eclampsia/eclampsia on bivariable analysis and were taken to multivariable analysis for controlling possible confounders were; marital status, family history of hypertension, history of pre-eclampsia, gravidity, pregnancy multiplicity, pregnancy planning, nutritional counseling during ANC, contraceptive use, alcohol and fruit intake during pregnancy.Even though attending ANC was significantly associated with preeclampsia on bivariable analysis, it was not included in the multivariable analysis due to collinearity.

Regarding to medical history related risk factors, multivariable analysis revealed that women who had previous history of preeclampsia had higher risk of pre-eclampsia. The odds of developing pre-eclampsia were 4 times higher for the women with history of pre-eclampsia comparing to their counter parts (AOR: 4.28, 95% CI: 1.61, 11.43). Primigravida was found to be risk factor for preeclampsia on the multivariable analysis. The odds of developing pre-eclampsia were 2.68 times higher in women with primigravida comparing to the women with multigravida (AOR: 2.68 95% CI: 1.38, 5.22). Women who had multiple pregnancies (twin) had higher risk of pre-eclampsia comparing to women with singleton pregnancy in the multivariable analysis (AOR: 8.22 95% CI: 2.97, 22.78). The multivariate analysis also revealed that receiving nutritional counseling during ANC contact was found to protect the women from pre-eclampsia. The risk of developing pre-eclampsia was lower among women who had received nutritional counseling (AOR: 0.22, 95% CI: 0.10, 0.48). Women who reported to have drunken alcohol during the pregnancy had also an increased risk of pre-eclampsia as compared to those women who did not drink alcohol in multivariable analysis (AOR = 3.97, 95% CI = 1.8, 8.75)(Table 2).

**Table 2:** Bivariable and multivariable analyses on determinants of pre-eclampsia/eclampsia among womenattended delivery services in selected public Hospitalsof Addis Ababa, Ethiopia, 2016

Variables	Cases	Controls	COR(95%: CI)	AOR(95%: CI)
	Number (%)	Number (%)		
Marital status				
Never married	13(13.67)	13(6.84)	2.27(1.01, 5.14)	0.76(0.19, 2.88)
Married/Living together	71(74.74)	161(84.74)	1	1
Others	11(11.58)	16(8.42)	1.56(0.69, 3.53)	1.34(0.43, 4.11)
Occupation				
Employed	26(27.34)	69(36.32)	1	1
House wife	47(49.47)	69(36.32)	1.81(1.01, 3.24)	1.72(0.83, 3.58)
Merchant	11(11.58)	41(21.58)	0.71(0.32, 1.59)	0.55(0.18, 1.64)
Student	11(11.58)	11(5.79)	2.65(1.03, 6.86)	2.23(0.67, 7.46)
Family history of hypertension				
No	83(87.4)	180(94.7)	1	1
Yes	12(12.6)	10(5.3)	2.6(1.08, 6.27)	2.24(0.67, 7.46)
History of preeclampsia				
No	27(71.05)	106(89.1)	1	1
Yes	11(28.95)	13(10.9)	3.32(1.34, 8.23)	4.28(1.61, 11.43) *
Gravidity				
Primigravida	57(60)	71(37.37)	2.5(1.52, 4.17)	2.68(1.38, 5.22) *
Multigravida	38(40)	119(62.63)	1	1
Pregnancy multiplicity				
Singleton	78(82.1)	181(95.3)	1	1
Twin	17(17.9)	9(4.7)	5.03(2.18, 11.62)	8.22(2.97, 22.78) **
Planned pregnancy				
No	21(22.11)	14(7.37)	3.57(1.72, 7.39)	2.1(0.68, 7.46)
Yes	74(77.89)	176(92.63)	1	1
Receiving nutritional counseling during ANC				
No	34(40)	23(12.6)	1	1
Yes	51(60)	160(87.4)	0.22(0.12, 0.4)	0.22(0.1, 0.48) **
Using contraceptives				
Non-users	32(33.68)	85(44.74)	1	1
Hormonal	56(58.95)	81(42.63)	1.84(1.08, 3.12)	1.07(0.52, 2.2)
Mechanical	5(5.26)	10(5.26)	1.32(0.42, 4.19)	0.87(0.15, 5.17)
Alcohol intake				
No	62(65.26)	169(88.95)	1	1
Yes	33(34.74)	21(11.05)	4.28(2.3, 7.9)	3.97(1.8, 8.75) *
Fruit intake				
No	15(15.79)	11(5.79)	3.05(1.34, 6.94)	1.57(0.47. 5.26)
Yes	80(84.21)	179(94.21)	1	1

*COR* crud odds ratio, *AOR* adjusted odds ratio, * *p*-value <0.05, **p-value <0.001

## Discussion

We conducted facility based unmatched case control study with the aim of assessing medical disease, obstetric history and behavioral determinants of preeclampsia/eclampsia. We found that primigravida, having positive history of preeclampsia in previous pregnancy, multiplicity of pregnancy and alcohol drinking during pregnancy as risk factor whereas nutritional counseling during antenatal care was a protective factorfor preeclampsia/eclampsia.

Concerning to medical disease, family history of hypertension was statistically significant with pre-eclampsia/eclampsia on bivariate analysis only (COR: 2.6 95% CI: 1.08, 6.27) but on the Dessie cross sectional study [10] revealed that women with positive family history of hypertension had 7 times odds of developing pre-eclampsia/eclampsia. The study subjects in Dessie were pregnant women who came for ANC follow up so, the difference could be due to the difference of study design and study subjects.

Multivariable analysis revealed that women who had previous history of pre-eclampsia had higher risk of pre-eclampsia/eclampsia (AOR: 4.28, 95% CI: 1.61, 11.43). This finding is consistent with the studies conducted in Iran [11, 12]. This implies women with history of preeclampsia/eclampsia on their prior history need to have a focus and could be an acceptable means of screening for pre-eclampsia, especially in limited resourced locations.

Primigravida was independently associated with pre-eclampsia/eclampsia in the multivariable analysis. This study is in agreement with studies conducted in Egypt [9], Nigeria [13], Uganda [14] and England (Krishnachetty, B. and F. Plaat: Managment of hypertensive disorders of pregnancy, unpublished) which declares primigravida was a risk factor for pre-eclampsia/eclampsia. Pre-eclampsia generally is considered a disease of the first pregnancy [15] which is due to the immunological incompetence seen in first pregnancy between fetoplacental and maternal tissues [16].

In this study, multivariable analysis revealed that multiplicity of pregnancy was independently associated with pre-eclampsia/eclampsia. Another studies conducted in Pakistan [17], India [18] and England also has a finding which is in light of this study. This is explained by women with multiple pregnancy obstetric conditions had a large placenta which results in decreased placental perfusion. The excess of placenta tissues could not be perfused adequately [19] comparing to the women with singleton pregnancy which lead both the mother and the fetus contribute to the risk of pre-eclampsia/eclampsia.

Multivariate analysis revealed that preeclampsia was lower on women who had received nutritional counseling comparing to the women who had not received nutritional counseling (AOR: 0.22 95% CI: 0.1, 0.48). This finding was in agreement with the study conducted in India [18] which showed received advice on pregnancy nutrition during ANC visit was a protective for pre-eclampsia/eclampsia. The protective factor of receiving nutritional counseling during ANC could be explained by the counseling on the healthy diet during pregnancy by the health workers which can be protective factor for pre-eclampsia.The content of nutritional counseling of the two hospitals include antioxidant and calcium rich dietary habits. Besides, there is a need for further studies to assess preventive nutritional factors for pre-eclampsia/eclampsia.

The multivariable analysis in this study indicates that alcohol intake during pregnancy was a significant risk factor for pre-eclampsia/eclampsia. This current finding is in contrast of the studies conducted in Uganda [14] and India [18] in which alcohol drinking during pregnancy had no significant association with pre-eclampsia/eclampsia. The cross-sectional study conducted in India, unlike the current study, was based on the women’s report of clinical manifestation for pre-eclampsia and were not diagnosed by physicians. More over the study subjects conducted in Uganda study were pregnant women who came for ANC follow up and labor ward with complications unlike to the current study which could be the reason for the difference.

This study could be seen in light of the limitations that the diagnosis for pre-eclampsia depend on the competency of obstetricians since this study used the diagnosed women. There might be introduced selection bias since cases were selected consecutively as they appear for diagnosis. Preeclampsia can be developed later on the postpartum period and the study subjects were not followed after discharge from hospitals. There might be also introduces recall bias for some information acquired using the memory of participants.Besides, the institutional study cannot represent the population.

## Conclusions

The results of this studysuggest that there are preventive and risk factors for preeclampsia. Some factors such as primigravida, twin pregnancy, having history of pre-eclampsia and drinking alcohol during pregnancy were the risk factors for the pre-eclampsia/eclampsia.Since identification of the riskfactors will enhance the ability to diagnose and monitor women likely to develop preeclampsia before the onset of disease for timely interventions and better maternal and fetal outcomes. Receiving nutritional counseling during pregnancy on ANC follow up were found that protective for the pre-eclampsia/eclampsia.It is recommended that health workers should use primigravida, preeclampsia in previous pregnancy and women with twin pregnancy factors as a screening tool for pre-eclampsia prediction and early diagnoses. Health care providers should give nutritional counseling including avoiding drinking alcohol for the pregnant women during their ANC visits. Further research is needed to understand more for the risk factors for pre-eclampsia/eclampsia particularly on dietary habits.

## Additional file

Additional file 1: English Version Questionnaire. (DOCX 33 kb)

## Abbreviations

ANC: Antenatal Care
AOR: Adjusted Odds Ratio
CI: Confidence Interval
COR: Crude Odds Ratio
GMH: Gandhi Memorial Hospital
HMIS: Health Management Information System
PE/E: Preeclampsia/Eclampsia
SD: Standard Deviation
ZMH: Zewditu Memorial Hospital

## References

[1] American College of Obstetricians and Gynecologists (2013). Hypertension in Pregnancy,Task force on hypertension in pregnancy. Obstet Gynecol.

[2] Green P.Update in the Diagnosis and Management of Hypertensive Disorders in Pregnancy. Michigan: Wayne State University School of Medicine; 2014.

[3] Al-Jameila N (2014). A brief overview of preeclampsia. J Clin Med Res.

[4] World health organization (2011). Recommendations for prevention and treatment of pre-eclampsia and eclampsia.

[5] Federal Democratic Republic of Ethiopia Ministry of Health (2010). Health sector development Programme IV 2010/11 – 2014/15.

[6] Federal Democratic Republic of Ethiopia Ministry of Health (2013). Maternal death surveillance and response (MDSR) technical guideline.

[7] Maternal and Child Health Integrated Program (MCHIP) (2011). Interventions for impact in essential obstetric and newborn care.

[8] JSI Research & Training Institute (2009). Addressing community maternal and neonatal health in Ethiopia. Report from National Scoping Exercise and National Workshop to increase demand, accesses and use of community maternal and neonatal health services.

[9] El-Moselhy EA, Khalifa HO, Amer SM, Mohammad KI, Abd El-Aal HM (2011). Risk factors and impacts of pre-Eclampsia: an epidemiological study among pregnant mothers in Cairo, Egypt. J Am Sci.

[10] Tessema GA, Tekeste A, Ayele TA (2015). Preeclampsia and associated factors among pregnant women attending antenatal care in Dessie referral hospital, Northeast Ethiopia: a hospital-based study. BMC Pregnancy and Childbirth.

[11] Direkvand-Moghadam A, Khosravi A, Sayehmiri K (2012). Predictive factors for preeclampsia in pregnant women: a unvariate and multivariate logistic regression analysis. Biochimica Polonica.

[12] Kashanian M (2011). Risk factors for pre-Eclampsia: a study in Tehran, Iran. Arch Iran Med.

[13] Guerrier G (2013). Factors associated with severe preeclampsia and eclampsia in Jahun, Nigeria. Int J Women’s Health.

[14] Kiondo P (2012). Risk factors for pre-eclampsia in Mulago hospital, Kampala, Uganda. Trop Med Int Health.

[15] Harutyunyan A (2009). Investigation of risk factors for preeclampsia development among reproductive age women living in Yerevan, Armenia: a case-control study.

[16] Sibai BM. Diagnosis and managment of gestational hypertension and pre-eclampsia. Obstet Gynecol. 2003;102(1):181–92. 10.1016/s0029-7844(03)00475-7 12850627

[17] Shamsi U, Saleem S, Nishter N. Epidemiology and risk factors of preeclampsia; an overview of observational studies*.* US Natl Libr Med Enlisted J. 2013;6(4):292-300.

[18] Agrawal S, Walia GK. Prevalence and Risk Factors for Symptoms Suggestive of PreEclampsia in Indian Women. J Women’s Health, Issues & Care. 2014;3(6). http://dx.doi.org/10.4172/2325-9795.1000169.

[19] Robert JM and Gammil HS, Preeclampsia: recent insights. American Heart Association. USA: Magee-Womens Research Institute; 2005.

