# Supplementary material for: Determinants of pre-eclampsia/Eclampsia among women attending delivery Services in Selected Public Hospitals of Addis Ababa, Ethiopia: a case control study
Source: BMC Pregnancy Childbirth. 2017 Sep 15;17:307. doi: 10.1186/s12884-017-1507-1 (PMC5603094; doi:10.1186/s12884-017-1507-1)
Supplement: Additional file 1: — English Version Questionnaire. (DOCX 33 kb) [file 12884_2017_1507_MOESM1_ESM.docx]

## **Questionnaire**

**Addis Ababa University**

**College of Health Science**

**School of Public Health**

**Questionnaire prepared to assess determinants of Preeclampsia/Eclampsiaamong Women Attending Delivery Services in Selected Public Hospitals of Addis Ababa, Ethiopiain Addis Ababa public Hospitals, 2015**

**Information sheet**

Hello. My name is _______________________________________ I am on behalf of

Teklit Grum, a staff of Addis Ababa University School of Public Health. He is conducting a research on “determinants of Preeclampsia/Eclampsia among Women Attending Delivery Services in Selected Public Hospitals of Addis Ababa, Ethiopia in Addis Ababa”. He has received permission from Institutional Review Board of the College of Health Science Addis Ababa University.

You have been selected to participate in this study as you attend birth in one of the public hospitals in Addis Ababa. Your contact information has been obtained from your medical record. Permission to collect your contact information has been received from the head of Hospital.

If you are willing to participate I will ask you some questions concerning your socio-demographic, medical illness, obstetric history and dietary habits during pregnancy. The interview last no more than 30 minutes.

Your participation in the study is voluntary. You may ask any questions during the interview, or skip any question you think is inappropriate and stop it at any moment you want with no further negative consequences. I really appreciate your participation in the current study.

Your participation in the study poses no risk for you. There will be no monetary benefits for you if you participate in this project. The information provided by you is of great value for investigation of determinants for preeclampsia/eclampsia development, which will be very helpful for science and/or for other women with similar disease.

The information you provided is fully confidential and will be used only for the study. If you want to talk to anyone about this research study you can contact [teklitgwg@yahoo.com](mailto:teklitgwg@yahoo.com)/teklitvip@gmail.com

At this time, do you want to ask me anything about the research?

Would you be willing to participate? Yes No

**Written Consent**

I am willing to take part in the study called preeclampsia/eclampsia.  I understand that the researcher from Addis Ababa University are hoping to assess determinants for preeclampsia/eclampsia. I understand that I will be asked about socio-demographic, medical illness, obstetric history and dietary habits during pregnancy. This study will take place in Zewditu Hospital and Ghandi Hospital in Addis Ababa and should take about 30 minutes of my time.

I am taking part because I want to.  I have been told that I can stop at any time, and if I do not like a question, I do not have to answer it.  No one will know my answers, including strangers, parents, and other children. It will be used only for the study.

Signature of the respondent: ______________ Date: _____________

Interviewer name: ______________________________________________

Signature: _________________ Date: ________________

Thank you very much for your participation.

**Status: 1. Case 0. Control**

**ID____________**

| 1. **Demographic and Socioeconomic factors** | | | |
| --- | --- | --- | --- |
| Serial number | Question | Response | Go to |
| 101 | How old were you at your last birthday? | ______________ in years |  |
| 102 | What is your ethnicity? | 1. Amhara 2. Oromo 3. Tigray 4. Gurage 5. Sidamo 6. Afar 7. Welaita 8. Others (specify)________ |  |
| 103 | What is your religion? | 1. Orthodox 2. Muslim 3. Protestant 4. Catholic 5. Others(specify)_________ |  |
| 104 | What is your occupation? | 1. House wife 2. Merchant 3. Government employee 4. Private employee 5. Students 6. Others (specify)______ |  |
| 105 | Please tell me what your marital status is? | 1. Never married 2. Married or living together 3. Divorced 4. Separated 5. Widowed |  |
| 106 | Have you ever attended school? | 1. Yes 2. No …………………………………. | 108 |
| 107 | What is the highest level of school you attended: | 1. 1. Primary 2. 2. Secondary 3. 3. Technical/vocational 4. 4. Higher |  |
| 108 | Tell me your monthly house hold income? | ______________ USD |  |
| 1. **Medical illness factors** | | | |
| 201 | Had any body from your family had or have hypertension? (check all that apply for first degree relatives only) | 1. Yes 2. No………………………………………. | 203 |
| 202 | If yes, who? | 1. Father 2. Mother 3. Sister 4. Brother |  |
| 203 | Had any body from your family had or have Diabetes mellitus? (check all that apply for first degree relatives only) | 1. Yes 2. No ………………………………………. | 205 |
| 204 | If yes, who? | 1. Father 2. Mother 3. Sister 4. Brother |  |
| 205 | Have you been told by a physician that you had or have Diabetes mellitus? | 1. Yes 2. No |  |
| 206 | Have you been told by a physician that you had or have asthma? | 1. Yes 2. No |  |
| 1. **Personal reproductive related factors** | | | |
| 301 | Have you been pregnant before this pregnancy? (Include all pregnancies that ended in life births, spontaneous or induced abortions, ectopic pregnancy and stillbirth as well) | 1. Yes 2. No ………………………………………. | 310 |
| 302 | How many have you been pregnant?  (including the current pregnancy) | ____________Times |  |
| 303 | What was the time intervalbetween previous and this pregnancy? | _____year____ month |  |
| 304 | Had the midwife or doctor told you that you had high blood pressure duringprevious pregnancy? | 1. Yes 2. No |  |
| 305 | Had the midwife or doctor told you that you had Gestational diabetes during previous pregnancy? | 1. Yes 2. No |  |
| 306 | Have you ever had history of abortion? | 1. Yes 2. No ……………………………………. | 308 |
| 307 | How many abortions do you had? | ____________Times |  |
| 308 | Have you ever given birth before? | 1. Yes 2. No …………………………………. | 310 |
| 309 | How many times have you givenbirth? | ____________Times |  |
| 310 | When you got pregnant, did you want to get pregnant at that time? | 1. Yes 2. No |  |
| 311 | What is the multiplicity of mother’s for the current pregnancy? | 1. Single 2. Twins ………………………………. | 313 |
| 312 | What is the sex of newborn for the current pregnancy? | 1. Male 2. Female |  |
| 313 | What is the sex of first bornfor the current pregnancy? | 1. Male 2. Female |  |
| 314 | What is sex of second bornfor the current pregnancy? | 1. Male 2. Female |  |
| 315 | Have you attend ANC clinic for this pregnancy? | 1. Yes 2. No …………………………………… | 318 |
| 316 | Number of ANC clinic visit | _______ times |  |
| 317 | Have you received nutritional advice during ANC | 1. Yes 2. No |  |
| 318 | Were you using modern contraceptive in the last 12 months before this pregnancy? | 1. Yes 2. No …………………………………….. | 320 |
| 319 | What type? | - 1. Pill   2. Injectable   3. Implant   4. Condom   5. IUCD |  |
| 320 | What is the type of maternal blood group?(check from medical chart) | 1. A 2. B 3. AB 4. O |  |
| 321 | RH factor(check from medical chart) | 1. Positive 2. Negative |  |
| 322 | What is the hemoglobin level of the mother in mg/dl? (check from medical chart) | _____ mg/dl |  |
| 1. **Pregnancy behavior factors** | | | |
| 401 | Were you smoking cigarette during the pregnancy? | 1. Yes 2. No ……………………………………. | 403 |
| 402 | How often? | 1. Daily:____cigarette per day 2. Sometimes:_____cigarette per week 3. I stopped smoking at______ weeks of that pregnancy |  |
| 403 | Were you drinking alcohol (Beer, Tala, Arekie/Katikala, Wine, Tej and others) during the pregnancy? | 1. Yes 2. No ……………………………………… | 405 |
| 404 | Which type? | 1. Beer 2. Tala 3. Arekie/Katikala 4. Wine 5. Tej 6. Others specify _______ |  |
| 405 | Were you drinking coffee during pregnancy? | 1. Yes 2. No…………………………………… | 407 |
| 406 | How often? | 1. Daily 2. ______ days per week 3. I have stopped at ______ weeks of gestational age |  |
| 407 | Were you eating fruit during pregnancy? | 1. Yes 2. No………………………… | 409 |
| 408 | How often? | 1. Daily 2. ______ days per week 3. I have stopped at ______ weeks of gestational age |  |
| 409 | Were you eating vegetables during pregnancy? | 1. Yes 2. No ……………………… | 411 |
| 410 | How often? | 1. Daily 2. ______ days per week 3. I have stopped at ______ weeks of gestational age |  |
| 411 | Were you doing physical exercise during pregnancy | 1. Yes 2. No ……………………….. | 413 |
| 412 | How often | 1. Daily 2. ______ days per week 3. I have stopped at ______ weeks of gestational age |  |
| 413 | Were you using traditional medicine during pregnancy | 1. Yes 2. No |  |

**THANK YOU**
